# Supplementary material for: The role of cochlear place coding in the perception of frequency modulation
Source: eLife. 2020 Sep 30;9:e58468. doi: 10.7554/eLife.58468 (PMC7556860; doi:10.7554/eLife.58468)
Supplement: Supplementary file 1. — (a) AM incoherence discrimination results. (b) Complex AM detection results. (c) Results with outlier subject included (n = 56) generally demonstrate the same trends as the main text, with the exception that the correlation between slow FM and slow AM detection thresholds was significantly different from the correlation between fast FM and fast AM detection thresholds (Z = 2.13, p=0.032, two-tailed), an effect that was not present with the outlier removed (see ‘Correlations between FM and AM detection’ in main text). (d) AM discrimination conditions. The units for all columns except Octave Separation are in Hz. [file elife-58468-supp1.docx]

**SUPPLEMENTARY INFORMATION**

**Supplementary File 1A.** AM incoherence discrimination results.

| **Effect** | **DF_effect_** | **DF_error_** | ***F*** | ***p*** | ***η_p_²*** |
| --- | --- | --- | --- | --- | --- |
| Frequency Separation | 1 | 19 | 72.3 | **< .0001** | .792 |
| Center Frequency | 2 | 38 | 38 | **< .0001** | .667 |
| Rate | 1 | 19 | 10.9 | **.004** | .365 |
| Frequency Separation*Center Frequency | 2 | 38 | 11.6 | **.0001** | .379 |
| Frequency Separation*Rate | 1 | 19 | 1.66 | .213 | .08 |
| Center Frequency*Rate | 1.51 | 28.7 | 16.6 | **< .0001** | .466 |
| Frequency Separation*Center Frequency*Rate | 2 | 38 | .841 | .439 | .042 |

**Supplementary File 1B.** Complex AM detection results.

| **Effect** | **DF_effect_** | **DF_error_** | ***F*** | ***p*** | ***η_p_²*** |
| --- | --- | --- | --- | --- | --- |
| Phase | 1 | 19 | 9.93 | **.005** | .343 |
| Frequency Separation | 1 | 19 | .964 | .339 | .048 |
| Center Frequency | 2 | 38 | 3.04 | .059 | .138 |
| Rate | 1 | 19 | 20.9 | **.0002** | .524 |
| Phase*Frequency Separation | 1 | 19 | .183 | .673 | .01 |
| Phase*Center Frequency | 2 | 38 | 25.2 | **< .0001** | .571 |
| Frequency Separation*Center Frequency | 2 | 38 | .029 | .972 | .002 |
| Phase*Frequency Separation*Center Frequency | 2 | 38 | 1.85 | .171 | .089 |
| Phase*Rate | 1 | 19 | 4.94 | **.039** | .206 |
| Frequency Separation*Rate | 1 | 19 | .235 | .633 | .012 |
| Phase*Frequency Separation*Rate | 1 | 19 | 2.68 | .118 | .123 |
| Center Frequency* Rate | 2 | 38 | 1.15 | .326 | .057 |
| Phase*Center Frequency*Rate | 2 | 38 | 7.58 | **.002** | .285 |
| Frequency Separation*Center Frequency* Rate | 2 | 38 | .523 | .597 | .027 |
| Phase*Frequency Separation*Center Frequency*Rate | 1.38 | 26.1 | 1.47 | .244 | .072 |

**Supplementary File 1C.** Results with outlier subject included (n=56) generally demonstrate the same trends as the main text, with the exception that the correlation between slow FM and slow AM detection thresholds was significantly different from the correlation between fast FM and fast AM detection thresholds (Z = 2.13, *p* = .032, two-tailed), an effect that was not present with the outlier removed (see “Correlations between FM and AM detection” in main text).

| **Variable X** | **Variable Y** | **Statistics** |
| --- | --- | --- |
| Absolute thresholds at 1 kHz | Low slopes | *r* = -.703, *p* < .0001, CI = [-.815, -.54] |
| Absolute thresholds at 1 kHz | High slopes | *r* = -.707, *p* < .0001, CI = [.545, .818] |
| Slow FM | Fast FM | *r* = .867, *p* < .0001, CI = [.782, .92] |
| Slow AM | Fast AM | *r* = .617, *p* < .0001, CI = [.423, .757] |
| Fast FM | Fast AM | *r* = .45, *p* = .0006, CI = [.212, .637] |
| Slow FM | Slow AM | *r* = .2, *p* = .068, CI = [-.066, .44] |

**Supplementary File 1D.** AM discrimination conditions. The units for all columns except Octave Separation are in Hz.

| **Octave Separation** | ***f_m_*** | **Center Frequency** | ***f_c1_*** | ***f_c2_*** | **TEN Lower Cutoff** | **TEN**  **Upper Cutoff** |
| --- | --- | --- | --- | --- | --- | --- |
| 2/3 | 2 | 500 | 396.9 | 630 | 471.9 | 529.7 |
| 4/3 | 2 | 500 | 315 | 793.7 | 471.9 | 529.7 |
| 2/3 | 20 | 500 | 396.9 | 630 | 471.9 | 529.7 |
| 4/3 | 20 | 500 | 315 | 793.7 | 471.9 | 529.7 |
| 2/3 | 2 | 1500 | 1190.6 | 1889.9 | 1415.8 | 1589.2 |
| 4/3 | 2 | 1500 | 944.9 | 2381.1 | 1415.8 | 1589.2 |
| 2/3 | 20 | 1500 | 1190.6 | 1889.9 | 1415.8 | 1589.2 |
| 4/3 | 20 | 1500 | 944.9 | 2381.1 | 1415.8 | 1589.2 |
| 2/3 | 2 | 7000 | 5555.9 | 8819.4 | 6607.1 | 7416.2 |
| 4/3 | 2 | 7000 | 4409.7 | 11111.8 | 6607.1 | 7416.2 |
| 2/3 | 20 | 7000 | 5555.9 | 8819.4 | 6607.1 | 7416.2 |
| 4/3 | 20 | 7000 | 4409.7 | 11111.8 | 6607.1 | 7416.2 |
